# Opportunities and challenges for digital morphology

**DOI:** 10.1186/1745-6150-5-45

**Published:** 2010-07-06

**Authors:** Alexander Ziegler, Malte Ogurreck, Thomas Steinke, Felix Beckmann, Steffen Prohaska, Andreas Ziegler

**Affiliations:** 1 Institut für Immungenetik, Charité-Universitätsmedizin Berlin, Campus Benjamin Franklin, Freie Universität Berlin, Thielallee 73, 14195 Berlin, Germany; 2 GKSS-Forschungszentrum Geesthacht, Institut für Werkstoffforschung, Max-Planck-Strasse 1, 21502 Geesthacht, Germany; 3 Zuse Institute Berlin, Takustrasse 7, 14195 Berlin, Germany

## Abstract

Advances in digital data acquisition, analysis, and storage have revolutionized the work in many biological disciplines such as genomics, molecular phylogenetics, and structural biology, but have not yet found satisfactory acceptance in morphology. Improvements in non-invasive imaging and three-dimensional visualization techniques, however, permit high-throughput analyses also of whole biological specimens, including museum material. These developments pave the way towards a digital era in morphology. Using sea urchins (Echinodermata: Echinoidea), we provide examples illustrating the power of these techniques. However, remote visualization, the creation of a specialized database, and the implementation of standardized, world-wide accepted data deposition practices prior to publication are essential to cope with the foreseeable exponential increase in digital morphological data.

**Reviewers:**

This article was reviewed by Marc D. Sutton (nominated by Stephan Beck), Gonzalo Giribet (nominated by Lutz Walter), and Lennart Olsson (nominated by Purificación López-García).

## Introduction

The digital era, which has so successfully transformed scientific work in various disciplines, has also begun to be implemented in biology [1], but has not yet found satisfactory acceptance in morphology. Although this discipline is clearly at the heart of biological understanding, its digital implementation today is still in its infancy. Almost ten years ago, C. Godfray [2] already identified a number of problems associated with comparative morphology, in particular the virtual absence of web-based taxonomic information. A further problem was emphasized by E. Pennisi [3], who pointed out that by using traditional morphological techniques "a complete tree of life is centuries away". However, it is now foreseeable that non-invasive imaging techniques, in particular because they offer the opportunity to conduct high-throughput studies and the suitability to analyze whole specimens in a non-destructive manner, together with the broader availability of the respective instruments, will change the way by which morphological data will be acquired and analyzed in the future. Based on these technical advances, many morphologists do in fact believe that a golden age of morphology has begun [4-6]. As we show here, the advent of non-invasive, digital imaging techniques offers a multitude of opportunities, but presents also numerous challenges which need to be discussed within the life sciences and bioinformatics communities.

## Discussion

### Non-invasive, high-throughput imaging of whole specimens

Within the last ten years, resolution and suitability of modern imaging techniques, among them confocal laser scanning microscopy (CLSM), synchrotron radiation micro-computed tomography (SRμCT), micro-computed tomography (μCT), and magnetic resonance imaging (MRI), have led to a considerable increase in morphological studies that generate entirely digital raw data [e.g. [7-10]]. Conventional histological or ultrastructural methods typically take days or weeks before they yield first results, and the necessary preparations alter or may even destroy the specimen under study. However, by applying fast digital scanning protocols, it has now become possible to implement a high-throughput approach using whole, intact specimens [11,12]. Although CLSM as well as SRμCT yield excellent image data at high resolutions, we will focus here on μCT and MRI because of (i) the broad availability of the respective instruments, (ii) the possibility to scan specimens within the size range of several centimeters in a standardized manner, and (iii) the feasibility of high-throughput studies involving dozens or even hundreds of specimens.

**Figure 1:**
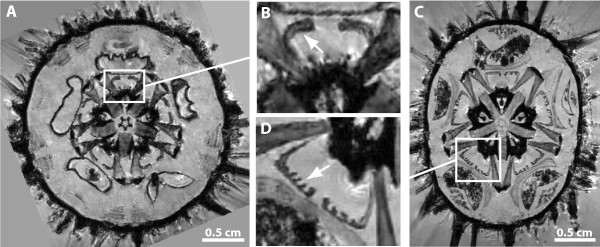
**Virtual horizontal sections of 2D MRI scans of whole sea urchins (A, C) reveal distinct shapes of protractor muscles in *Paracentrotus lividus *(B) and *Echinometra mathaei *(D) (arrows)**. Magnetic resonance imaging was carried out in Berlin, Germany using a high-field MRI scanner with a 7 T super-conducting electromagnet (Bruker Biospin GmbH, Ettlingen, Germany). Image processing of the ~10 MB large raw image datasets was carried out using ImageJ 1.42q (NIH, Bethesda, USA) and its Volume Viewer plug-in on a standard office PC. The sea urchin (Echinodermata: Echinoidea) species shown here were collected in the wild (*Paracentrotus lividus*) or taken from a museum collection (*Echinometra mathaei*, NHM 1969.5.1.61-75).

**Figure 2:**
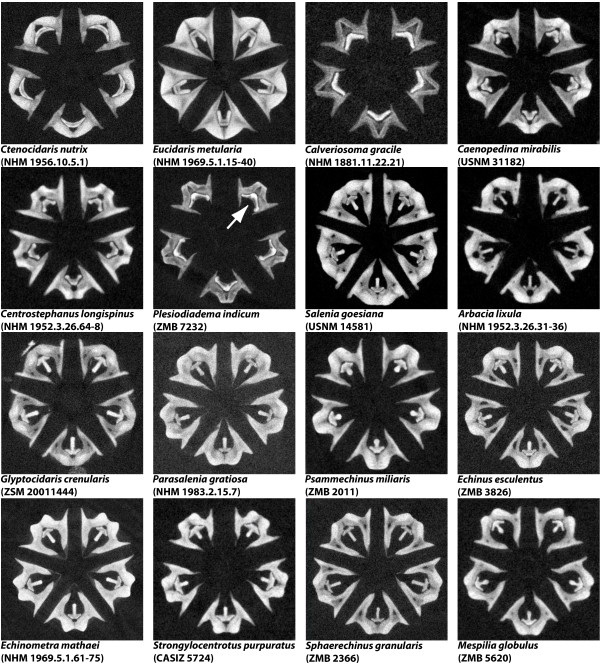
**Virtual horizontal μCT sections through Aristotle's lantern, the sea urchin feeding apparatus - phylogenetically representative selection of 16 out of almost 80 sea urchin species scanned by us using μCT (individual isotropic dataset resolutions ranging from 9 - 24 μm)**. The arrow depicts an asymmetrical tooth in *Plesiodiadema indicum *(Aspidodiadematidae). μCT was carried out at the GKSS outstation on the DESY site in Hamburg, Germany using an X-ray tube tomography system (GE Sensing & Inspection Technologies, Wunstorf, Germany). Image processing was carried out using Amira 5.2 (Visage Imaging GmbH, Berlin, Germany). The ~20-25 GB large raw image datasets were analyzed on a standard office PC using a remote visualization software environment (HP Remote Graphics Receiver 5.3.0: Hewlett-Packard Development Company, Palo Alto, USA). The office PC was connected via Internet (100 Mbit/s) to an HP XC/SVA visualization cluster (1× HP ProLiant DL785 with 32 AMD cores/256 GB RAM, and 8× HP xw8600 workstations, all equipped with dual NVIDIA Quadro FX5600 GPUs) at the Zuse Institute Berlin, Germany. Not to scale.

Whereas μCT is primarily used to display hard tissue, MRI is particularly suited for soft tissue studies. Furthermore, MRI can be used for longer three-dimensional (3D) as well as for much shorter two-dimensional (2D) scanning protocols. The latter permit a considerable reduction in scan time: ~10 min as opposed to ~12 h or even more [10], although this is accompanied by a reduction of resolution in the third dimension [see e.g. reference [13] for protocols]. Using this approach, the 2D scanning of over 50 species of sea urchins (Echinodermata: Echinoidea) at 79 μm in-plane resolution in just two days, for example, has allowed us to discover the presence of previously unrecognized specialized muscles within the feeding apparatus of selected taxa (Fig. 1). When μCT is applied, the times necessary for scanning whole specimens in 3D can be shortened considerably (to about 15 min for low- or about 2 h for high-resolution scans) and in general much higher resolutions can be achieved as compared to MRI [14]. Using this approach, we have scanned almost 80 selected sea urchin species in less than three weeks at isotropic resolutions ranging from 9 to 24 μm. Among other previously unknown characters, these analyses reveal the presence of asymmetrical teeth in a taxon of deep-sea echinoids, the Aspidodiadematidae (Fig. 2).

A number of recent technological advances demonstrate that the full potential of current non-invasive imaging techniques has presumably not even remotely been exploited [15-17]. μCT and MRI in particular will profit from research carried out in the near future because of the wide-spread use of non-invasive imaging techniques in human diagnostics. Apart from instrument-dependent improvements in image acquisition, the use of inexpensive contrast agents, e.g. iodine or manganese, for staining of soft tissues will further increase the applicability of μCT and SRμCT [18] as well as MRI [19]. Although some contrast agents need to be applied *in vivo *(e.g. manganese), others have successfully been used *in vitro *(e.g. gadolinium, iodine), both on freshly fixed as well as on century-old museum specimens. Ideally, museum material should be scanned without the use of any contrast agent, and the feasibility of this approach has already been demonstrated for studies using μCT [20] as well as MRI [10].

The potential, especially of μCT, to visualize complex structures or features that could not be analyzed using conventional techniques has been utilized successfully in a number of morphological studies over the last years. This is particularly obvious in palaeontological studies, including analyses of specimens embedded in amber [21] as well as studies on fossilized specimens [22-24]. However, it should not be forgotten that μCT and MRI in particular currently cannot compete with light and electron microscopy in terms of resolution. Differential stainings for histological sections are a further bonus of the latter techniques and it is principally possible to generate digital tomographic data from consecutive histological or ultrastructural sections [25]. Nevertheless, the non-destructive and high-throughput analyses that are the most prominent features of MRI and μCT must be regarded as so advantageous that conventional procedures and modern scanning techniques should be seen as complementing and not as competing approaches. The combined use of various imaging procedures, termed 'multimodality', has recently been implemented in a number of studies on vertebrate [26], arthropod [27], and echinoderm [28] taxa.

### Accelerated raw image data acquisition and increased taxon sampling

Drastically shortened scan times allow an increased number of specimens to be studied, thereby providing morphologists with the possibility to accomplish a broad taxon sampling for phylogenetic or comparative morphological analyses. This more exploratory *modus operandi *(in contrast to a focused, hypothesis-driven approach) is likely to yield novel insights into form as well as function and, as shown in an exemplary fashion in case of our research on sea urchin morphology, will result in many new questions and hypotheses (Figs. 1, 2). The data emerging from such studies can then be used to fine-tune further research using classical preparative techniques. On the other hand, the time that can be devoted to the analysis of a single species will increase by omitting the lengthy, conventional preparative procedures that have dominated morphological studies for hundreds of years. Apart from freshly collected specimens, μCT and MRI have successfully been applied to the study of museum specimens, incl. type material [29,30]. Although large-scale comparative morphological analyses based on advanced digital imaging modalities have so far been exploited only in a handful of studies [10,14,28], the predicted increase in digital data necessitates advances primarily in two areas: the remote visualization of complex data and the creation of a universally accepted morphological database.

### Remote visualization of morphological data

Since a linear increase in the resolution of image data entails a cubic increase in data size, it is mandatory to devote considerable attention to adequate archiving and computation systems as well as to advanced software for the purpose of 3D visualization and interactive viewing. MRI raw datasets of whole specimens [conventionally in the megabyte (MB, 10^6 ^bytes) range] can usually still be handled with current office PCs, but μCT or SR μCT datasets [usually in the gigabyte (GB, 10^9 ^bytes) range] cannot.

We therefore propose the analysis and visualization of morphological image data to be carried out using a data center setup that encompasses large storage resources and data processing capabilities with suitable large memory and powerful graphics cards [31]. This facility could be accessed, for instance, by remote visualization software environments, so that the manipulation of raw morphological data becomes possible on virtually any PC with Internet access (please refer to the legend of Fig. 2 for a more detailed explanation of our setup). In contrast to local visualization, i.e. using solely the resources of the user's computer, remote visualization will facilitate keeping abreast with the constant technical advances in computation and visualization by focusing investments in infrastructure on a limited number of facilities. Recent developments in other scientific disciplines have shown that only the larger institutions can afford to maintain the human as well as the technical resources that are necessary for the complex computation, archiving, and visualization processes involving large amounts of data. Certainly, such a setup would find acceptance only if the data deposited would be managed professionally, assuring their constant availability both for download and remote visualization. For example, several scientific funding organizations (such as the Helmholtz-Gemeinschaft in Germany) demand that raw data should be accessible for a minimum of ten years, in turn requiring backup systems that simply cannot be maintained at smaller institutions.

### The urgent need for a standardized morphological database

Specialized databases serving other biological disciplines are universally accepted as indispensable tools for obtaining a better understanding of biological facts (e.g. NCBI GenBank [32] or RCSB Protein Data Bank [33]). According to Foster [34], a community's shared understanding will no longer exclusively be found in the literature, but in the future also in databases. Unfortunately, the few publicly available digital morphological data are currently scattered throughout a number of independent databases (e.g. BIRN [35], DigiMorph [36], EOL [37], Morphbank [38], MorphDBase [39], MorphoBank [40]) that are funded by different institutions with diverging interests as well as financial capabilities. Although a considerable number of non-invasively acquired datasets have already been deposited online [e.g. [35,36,39]], the currently available morphological databases suffer either from limited input or from a restricted focus. For example, we have deposited parts of our raw image data on DigiMorph (μCT data) as well as on MorphDBase (MRI data), which makes a direct comparison of different datasets obtained from the same specimen unnecessarily complicated. Moreover, in the case of all morphological databases, the interactive visualization, i.e. the real-time manipulation of raw image datasets, as well as the download of these datasets are currently not available.

The creation and management of a universally accepted, morphologically oriented database is, however, not trivial. This is primarily a consequence of two independent factors: the datasets to be deposited require substantially more data storage capacity than, for example, sequence data, while, on the other hand, morphological data *per se *suffer from the current lack of a standardized ontology - this has been referred to as the "linguistic problem of morphology" [41]. Since raw image datasets nowadays may easily reach the GB range, it is of the utmost importance to solve the problem of their storage, archiving, and management. To further illustrate this point, our analyses of almost 80 sea urchin species by μCT have so far resulted in gathering of ~3 terabytes (TB, 10^12 ^bytes) of raw image data. It is principally possible to deal successfully with such amounts of data as shown by the Large Hadron Collider at CERN, Geneva, which has been designed to produce several petabytes (PB, 10^15 ^bytes) of raw data per year, but provides also the facilities that are essential for their adequate storage and analysis.

It is obvious that distinct imaging techniques produce different types of raw image data and that the implementation of data and metadata standards will be of critical importance for the success of a morphological database. However, an agreement on data standards could still be years away, and we therefore believe that by initially restricting the datasets to be deposited to MRI and μCT datasets, the repository could get starting right away, in turn leading to the standardization of all future datasets to be deposited by setting a strong standard.

Whether a centralized (e.g. NCBI GenBank [32], RCSB Protein Data Bank [33]) or a federated repository [42] that unites the already existing individual morphological databases would better serve the needs of the morphological community cannot currently be decided and is a matter that should be discussed within the life sciences and bioinformatics communities.

### Necessity for enforced data deposition

In addition, for a morphological repository to be successful - desirably with the possibility to remotely visualize raw image data on virtually any imaging device connected to the Internet, non-invasive imaging techniques and the drastic increase in digital morphological data acquisition furthermore necessitate the implementation of mandatory data deposition prior to publication. Enforced data deposition has long been accepted, for example, by structural biologists [33]. Although the preparation of morphological data for deposition in the context of a publication may take several days and is initially likely to lack popularity among the morphological community, its numerous advantages, in particular the general accessibility of the data and the resulting data transparency, clearly justify the effort.

**Figure 3:**
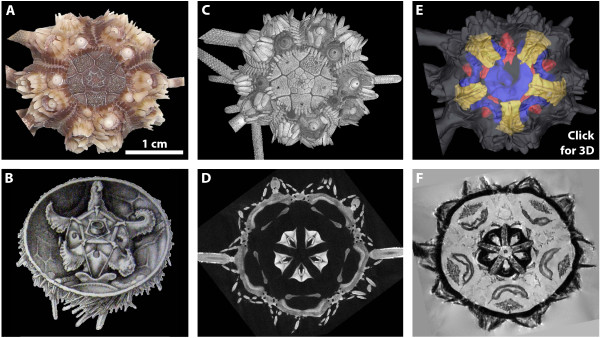
**Various traditional and digital morphological visualization techniques, shown in an exemplary fashion using cidaroid sea urchins (Echinoidea: Cidaroida)**. **A **Habitus of a museum specimen of *Eucidaris metularia *(NHM 1969.5.1.15-40), aboral view. **B **Historical drawing of the internal anatomy of *Cidaris cidaris*, a closely related species - modified after Stewart [47]. **C **Volume rendering of the external anatomy of the specimen shown in **A**, based on a ~25 GB large μCT dataset with 13.91 μm isotropic resolution. **D **Virtual horizontal section of the μCT dataset at the level of Aristotle's lantern (see also Fig. 2). **E **Surface rendering of the external and internal anatomy of the specimen shown in **A **based on a ~100 MB large 3D MRI dataset with 81 μm isotropic resolution. **F **Virtual horizontal section of the MRI dataset at the level of Aristotle's lantern, digestive tract, and gonads. By clicking anywhere onto this figure, an interactive, partially labeled 3D model of the analyzed species will open (requires Adobe Acrobat Reader 8.0 or higher, see [10,43,44,46] for detailed information regarding 3D modeling and labeling). The museum specimen of *Eucidaris metularia *was photographed using a digital camera with 7.2 megapixel resolution (Exilim: Casio Computer Co., Tokyo, Japan). 3D visualization was carried out using volume rendering in VG Studio Max 2.0 (**C**) and threshold-based as well as manual segmentation followed by surface rendering in Amira 5.2 (**E**).

As in other scientific fields that have already implemented digital data management procedures and raw data repositories, new areas of study will emerge in morphology as well, such as data mining or systematic combination of multimodally acquired raw image datasets. In other disciplines (e.g. astronomy or genomics), such novel approaches have led to a considerable increase in knowledge. Furthermore, the public database which we envisage here will also permit genotype/phenotype correlations, thereby serving as a reference for studies in all of the life sciences. In addition, digitally acquired data permit novel ways to communicate results, e.g. in the form of publication-embedded multimedia content (Fig. 3) [10,43-46].

Apart from these purely scientific considerations, a morphological database should additionally serve as a single point of contact for the interested layman as well as for students. We therefore believe that the advent of the digital era in morphology promises to lead to an improved visibility of this fundamental biological discipline on the whole. Furthermore, we are convinced that enforced data deposition is of crucial importance for the success of a morphological database. The morphological community should therefore rapidly seek the combined advice from database experts and leading journals in the field to implement this desirable feature.

## Conclusions

Critically, the future availability of all digital data deposited in a morphological repository should be guaranteed by a long-term (decades, not years) financial commitment by national or international scientific funding organizations. The benefits of this novel facility would by far outweigh the costs, since a standardized repository for digital morphological data will boost intra- and interdisciplinary investigations. We fully agree with Budd & Olsson [4] that "there has never been a better time to study morphology", but the morphological community must act now in order to catch up with disciplines that benefit already greatly from deposited digital data.

## Abbreviations

CASIZ: California Academy of Sciences Invertebrate Zoology, San Francisco, USA; CLSM: confocal laser scanning microscopy; MRI: magnetic resonance imaging; μCT: micro-computed tomography; SRμCT: synchrotron radiation micro-computed tomography; NHM: The Natural History Museum, London, UK; USNM: The National Museum of Natural History, Washington D.C., USA; ZMB: Museum für Naturkunde, Berlin, Germany; ZSM: Zoologische Staatssammlung München, Germany.

## Competing interests

The authors declare that they have no competing interests.

## Authors' contributions

AlZ conducted the scanning experiments, accomplished 3D visualization, and wrote the article. MO and FB developed and tested the protocols for μCT scanning of whole sea urchin specimens. TS and SP implemented and set up the remote visualization environment and provided support with the 3D visualization of large raw image datasets. AnZ wrote the article. All authors read and approved the final version of the manuscript.

## Reviewers' comments

### Reviewer's report 1

Mark D. Sutton, Dept. Earth Science & Engineering, South Kensington Campus, Imperial College, London, United Kingdom (nominated by Stephan Beck, UCL Cancer Institute, University College London, UK)

The authors of this contribution make a case for the importance of digital morphology as a tool for biologists; this case is not novel, although its reiteration here is to be welcomed. It might perhaps be strengthened through the provision of more examples beyond the authors' own research. The manuscript might be also profitably deal more explicitly with the importance of tomographic methods in a palaeontological context. Tomographic techniques provide not simply a faster and more convenient means of extracting morphological data, but for certain fossils the only feasible means of imaging morphology at all. They thus enable the extraction of previously unavailable data; this is doubly important in a field where genetic and other non-morphological information is essentially absent.

### Author's response

*In the revised version of our manuscript, we now cite a number of review articles that may serve as valuable starting points for the general reader (e.g. refs*. [7-12]). *Furthermore, we refer to the particular usefulness of non-invasive imaging techniques in palaeontology and have expanded this section in our manuscript*.

The three-fold call for (a) a centralised repository for digital morphology data, (b) enforced data-deposition, and (c) remote visualisation tools are also to be welcomed. However I would stress that all three of these 'pillars' would need to rest on the foundation of standardised data and metadata formats for morphological information. Morphological datasets come in many forms, from the isotropic CT tomographic stacks, through colour optically-captured grinding/sawing/microtomy stacks, to point-cloud laser-scanning data and photogrammetry-generated textured polygons. Interpretative layers in many cases are complex, requiring a flexible mark-up system to capture all desired information. Developing data standards and visualisation software to cover all possibilities is not a trivial task; software tools to aid workers in translating existing datasets into a new standardised format would also likely be required. The authors suggest that a centralised database would in itself act as a driver towards a resolution of these sorts of problems; I suspect that this would only be the case if the data standards and relevant software were of a high enough initial quality to overcome the reticence of the community. None of these problems are insurmountable or in any way detract from the desirability of a remotely-accessible comprehensive database of virtual morphology, but I feel they have been glossed over in the manuscript.

### Author's response

*In the revised version of the manuscript we discuss the problems that the morphological community faces with regard to data and metadata standardization. However, we would like to stress that in our eyes these issues will probably take years until they can be successfully dealt with. Because of the foreseeable exponential increase in digital morphological data in the years to come we nonetheless advocate the fast implementation of a morphological repository, suggesting that initially only datasets that can easily be standardized (e.g. MRI- or μCT-based) should be uploaded, which in turn would force other techniques to adapt to these standards*.

A more minor point related to the authors' enthusiasm for remote visualisation; while there are logistical benefits to this approach, there are also practical benefits to local visualisation, which can be accomplished with ease on relatively modest systems with sufficiently well optimised models and visualisation software, does not require high-bandwidth connections, and provides other practical benefits besides. These approaches are not of course mutually exclusive, and an ideal system would provide for both remote visualisation and the standardised packaging of morphological information for local visualisation and mark-up.

### Author's response

*We agree with the reviewer that a combined approach would be desirable. Therefore, raw image datasets should be available both for remote visualization as well as download. The constant advance in computer technology will sooner or later permit local visualization of datasets that currently can only be visualized on a standard PC by accessing remote visualization environments*.

### Reviewer's report 2

Gonzalo Giribet, Department of Organismic and Evolutionary Biology, Harvard University, Cambridge, MA, USA (nominated by Lutz Walter, Deutsches Primatenzentrum, Göttingen, Germany)

This article shows how some of the newest techniques in obtaining morphological data suffer from the challenge of dealing with large amounts of digital information. It comes in at the right time since, as illustrated by the several examples provided, there are large data sets of digital images being generated in many disciplines of biology and other sciences. The article is therefore of general interest for many scientists. I don't see any important issues with it and can be published with just taking into account a few minor comments/suggestions.

In page 6 the authors rank the problem of storing data above what has been called the "linguistic problem of morphology" but I do not see why these two problems need to be compared or ranked. Both may be equally important and in fact they are independent. Or maybe I did not understand what this paragraph really meant?

### Author's response

*We consider both problems to be independent from each other as well and have amended this section of the manuscript for clarity. We believe that, similar to the problem of data and metadata standardization, the creation of a morphological data repository should not be postponed by trying to achieve the ultimate goal of solving the problems associated with the creation of a standardized ontology*.

Page 5: Where it says "the predicted increase in digital data..." it may be better to say "the predicted increase in acquisition of digital data..."

NHM is the acronym for "The Natural History Museum", not simply "Natural History Museum"

Acknowledgements. "For generous supply with..." should be "for generously supplying..."

### Author's response

*We have changed the manuscript accordingly*.

### Reviewer's report 3

Lennart Olsson, Institut für Spezielle Zoologie und Evolutionsbiologie mit Phyletischem Museum, Jena, Germany (nominated by Purificación López-García, Université Paris Sud, Paris, France)

In this paper Alexander Ziegler and his co-authors point out that technical advances in non-destructive methods to document the internal morphology of organisms, such as X-ray computer tomography and MRI, in combination with 3D-reconstruction and visualizaton techniques, are ushering in a revolution in fields like anatomy, comparative morphology and palaeontology. They use examples from their own research on sea urchin morphology to show some of the advantages of these techniques. The authors discuss the problem of data storage and accessibility, and suggest that a specialized database be created, analogous to data banks for molecular sequence data. The paper will be of interest to researchers using, or planning to start to use, these techniques.

The main problem with the paper is the lack of originality. I would like to see more emphasis on what the authors feel is the unique contribution of their paper in the revised version. It could be argued that other authors have already made the same points in other papers, and why then publish another one?

### Author's response

*Our contribution is not intended to be a research paper, but a comment and to stimulate an ongoing discussion. The unique contribution of this manuscript is, however, that we introduce the demand for enforced data deposition practices in morphology and provide examples of how this could be achieved. The current lack of a widely accepted and universally accessible morphological repository shows that although in the past articles might have raised similar ideas, their implementation has clearly not been successful. However, the advent of high-throughput techniques necessitates action now in order to cope with the foreseeable drastic increase in digital morphological data*.

Other than this criticism, I have few complaints about the paper. One is that the authors mention in passing that tissues can be stained using "inexpensive contrast agents, e.g. iodine" (page 4). Staining and staining agents is an important topic and should be eleborated in some detail in the paper. Brian Metscher has shown that the choice of contrast agent can be important for visualizing soft tissues in vertebrates. His papers are mentioned (number 15 and 16 in the list of references) but this aspect is not discussed.

### Author's response

*In the revised version of the manuscript we now discuss staining techniques in greater detail*.

Details that need to be fixed include to explain what "GB domain" is (page 6), and to write "AlexanderZ" and "AndreasZ" as Alexander Z and Andreas Z on page 8.

### Author's response

*We have changed the manuscript accordingly*.
